# CD155 promotes radioresistance and malignancy of esophageal cancer by regulating Hippo-YAP pathway

**DOI:** 10.1007/s12672-022-00515-z

**Published:** 2022-06-29

**Authors:** Huixian Xin, Yuchen Liu, Pengxiang Chen, Tianwen Yin, Meijie Wang, Tianyu Liu, Zhihua Wen, Yufeng Cheng

**Affiliations:** 1 Department of Radiation Oncology, Cheeloo College of Medicine, Qilu Hospital, Shandong University, 250012 Jinan, Shandong China; 2 Department of Radiation Oncology, Shandong Cancer Hospital, and Institute, Cheeloo College of Medicine, Shandong University, 250012 Jinan, Shandong China

**Keywords:** Esophageal Cancer, CD155, Radiosensitivity, Carcinogenesis, Hippo Pathway, YAP

## Abstract

The expression of CD155 has been observed to increase in various human cancers, but its role in the development of esophageal cancer (EC) is unclear. Radiotherapy is one of the primary therapeutic options for EC. However, radioresistance is still a severe issue in EC treatment. In this study, Oncomine database mining, immunohistochemistry, and survival analysis showed that higher expression of CD155 in patients with EC than in healthy controls. In vitro and in vivo, we found for the first time that irradiation increased the expression of CD155 in EC cells. CD155 knockdown inhibited cell proliferation and migration and tumor formation, and significantly increased radiosensitivity in EC. The in vivo model with high CD155 expression significantly promoted the proliferation and migration of EC cells. Furthermore, increased CD155 expression was associated with poor prognosis in patients with EC. CD155 regulated the Hippo-Yap pathway, influencing cell proliferation and migration. Therefore, CD155 is essential for the proliferation, migration, and radioresistance of EC. CD155 inhibition may be a viable strategy for improving radiation treatment efficacy in individuals with EC.

**Supplementary Information:**

The online version contains supplementary material available at 10.1007/s12672-022-00515-z.

## Introduction

Esophageal cancer (EC) is the seventh globally most frequent disease and the sixth leading cause of cancer-related mortality [1]. Ionizing radiation(IR) is a traditional and effective method for treating cancer [2, 3], playing a significant role in therapeutics of malignant tumors such as esophageal squamous cell carcinoma (ESCC) [4], lung cancer, and breast cancer [5, 6]. Most EC patients locally progress upon diagnosis, with lengthy esophageal lesions and extensive lymph node invasion. Large prospective phase 3 studies have ruled definite chemoradiotherapy (dCRT) as the primary treatment option for these patients [7, 8]. However, the regulation for tumor radiosensitivity is challenging in radiotherapy. Therefore, the search for new molecules and mechanisms to increase radiosensitization in EC is urgently needed.

CD155 is an adhesion molecule involved in various physiological and pathophysiological processes. It belongs to the immunoglobulin superfamily and is also known as Necl5 [9]. It has four splice isoforms: α, β, δ, and γ. The α isoform contains an immunoreceptor tyrosine inhibitory motif (ITIM) [10], which provides a structural basis for CD155 signaling and tumor cell-intrinsic biology. Recently, CD155 was found to be highly expressed in different tumors [11, 12] and involved in signal transduction [13], cell proliferation [14], adhesion [15], migration [16–18], and immune response control [19–21] through its ITIM recruitment tyrosine phosphatase 2 (SHP-2). It is also associated with tumor progression. Being differentially regulated in several malignancies, CD155 holds considerable therapeutic promise as a biomarker and may be a new target molecule for cancer therapy.

In this study, we performed a comparative expression analysis of CD155 between tissue samples from patients with EC and control tissue samples. We found that higher CD155 expression is associated with a poor prognosis in patients with EC. Furthermore, we addressed CD155 cellular function either by knocking down or overexpressing CD155, both in vivo and in vitro.

## Materials and methods

### Cell lines

The human esophageal squamous cell carcinoma (ESCC) cell lines Eca109 (CCTCC, Wuhan, China) and Kyse510 (CCTCC, Wuhan, China) were cultured in RPMI 1640 medium (Gibco, USA) supplemented with 10% fetal bovine serum (Gibco), penicillin (100 U/ml), and streptomycin (100 g/ml). HEEC cell lines were generously provided by Renchang Zhao from the Thoracic Surgery team of Qilu Hospital and cultured in DMEM medium. Eca109 and Kyse510 cells were transfected with recombinant lentiviruses and positive clones were selected with puromycin (4 g/ml). Quantitative RT-PCR (qRT-PCR) and western blot analyses were used to assess the efficacy of lentiviral transfection. CD155 overexpression lentiviruses (NM 006505) were obtained from Shanghai Genechem Co., Ltd. CD155 gene knockdown lentiviruses (LPP-CS-HSH058084-LVRU6GP-01-200) were obtained from GeneCopoeia (Inc, Rockville, MD, USA. The symbols OE- and sh- means overexpression and knockdown, respectively. OE-NC, control group for overexpression; OE-CD155, overexpression of CD155; sh-NC, control group for knockdown; sh-CD155 knockdown of CD155.

### Clinical samples and information

A paraffin-embedded EC tissue chip (Catalogue no. Esc968) was purchased from Shanghai Superbiotek Pharmaceutical Technology Co., Ltd. (Shanghai, China).

### qRT-PCR

RNA*fast200* (Cat no. 20010, fastagen) was used to purify the total RNA from cells according to instructions from the manufacturer. qRT-PCR was conducted with SYBR Green Premix Pro Taq HS qPCR Kit (AG11701, ACCURATE BIOTECHNOLOGY, Hunan, China) according to instructions from the manufacturer and using the Bio-Rad Single-Color Real-Time PCR system (Bio-Rad, Hercules, California, USA). The primers (Accurate Biology, China) were designed and synthesized as follows: CD155: 5’-GCGTAGAGGATGAAGGCAACT-3’ (forward primer); CD155: 5’-CAAGCACTCGGAGCCAGATAT-3’ (reverse primer); GAPDH: 5’-GCACCGTCAAGGCTGAGAAC-3’ (forward primer); GAPDH: 5’-TGGTGAAGACGCCAGTGGA-3’ (reverse primer). Relative CD155 expression was calculated using the 2-∆∆CT method.

### Western blot

Total protein extract was isolated from cells lysed in pre-cooled RIPA buffer with protease inhibitors (BestBio, China), according to instructions from the manufacturer. Protein concentration in each extract was determined using a BCA protein assay kit (Beyotime Biotechnology, Wuhan, China). Proteins from the extract were separated according to size in SDS-PAGE acrylamide gel (10–15%) and subsequently transferred to a Poly (vinylidene fluoride) (PVDF) membrane (Millipore). After protein transfer, the membrane was blocked using 5% non-fat milk or bovine serum albumin for 2 h. The blocked membrane was incubated overnight with the primary antibodies (Table 1) at 4 °C. The membrane was then incubated with secondary anti-rabbit or anti-mouse antibodies (ZSGB-BIO) at room temperature for 2 h. Protein bands in the membranes were detected using an ECL kit (Millipore). Images of the blots were obtained using a CCD imager (Tanon-5200, Tanon Science & Technology).

**Table 1:** Antibody used in the study

Antibody	Catalog no./supplier
Recombinant Anti-Poliovirus Receptor/PVR antibody [EPR22672-151]	ab267788/ abcam
LATS1 (C66B5) Rabbit mAb	3447T/ CST
**Phospho-LATS1 (Thr1079) (D57D3) Rabbit mAb**	**#8654/ CST**
Phospho-YAP rabbit mAb	13308T/ CST
YAP (D8H1X) XP Rabbit mAb	14074T/ CST
Phospho-TAZ (Ser) (E1 × 9 C) Rabbit mAb	59,971 S/ CST
Anti-TAZ antibody	ab224239/ abcam
Anti-GAPDH Rabbit Polyclonal Antibody	A0102/ Abbkine
**HRP Conjugated Goat anti-Rabbit IgG Goat Polyclonal Antibody**	**HA1001/ HuaBio**
**Rabbit IgG**	**HA1002/ HuaBio**
Phospho-Stat3 (Tyr705) (D3A7) XP® Rabbit mAb	#9145/ CST
STAT1 (phospho Y701)	ab30645/ Abcam
Phospho-mTOR (Ser2448) (D9C2) XP® Rabbit mAb	#5536/ CST
Phospho-Akt (Ser473) (D9E) XP® Rabbit mAb	#4060/ CST
Phospho-ERK1/2 (Thr202/Tyr204) Antibody	# AF1015/ Affinity
Anti-CDK6 antibody	ER40101/ HUABIO

Data was evaluated using the Bliss independence model

### Cell viability assays

A cell counting kit-8 assay (CCK8; *Bioss* company, China) was used to evaluate the proliferation of Eca109 and Kyse510 cells. *Cells* (2000/*well*) *were seeded into 96*-*well plates*. After incubation for 0, 24, 48, 72, 96, and 120 h at 37 °C, *CCK8* assays were conducted according to instructions from the manufacturer. The optical density (OD) of each well was measured using an Infinite M200 PRO microplate reader (Tecan) with the 450 nm wavelength.

### Wound-scratch assay

A wound-scratch assay was performed on Eca109 and Kyse510 cells grown to confluence. Cells were seeded in six-well plates and cultured in serum-free culture medium. When the cells reached 100% confluency, a scratch line over the cell layer was made using a gun head perpendicular to the plate and washed twice with PBS to remove the floating cells. Gaps were observed and photographed at 0 and 24 h after scratching. The cell free area of the scratch was calculated using ImageJ software. The percentage of scratch healing was quantified as: (0 h scratch area-24 h scratch area)/0 h scratch area × 100. For each experiment, at least three scratched fields were recorded, and all wound-scratch assays were performed in triplicate (×10 magnification).

### Transwell assay

Equal numbers of cells from different groups were first grown into transwell chambers (3422, Corning, NY, USA), which were incubated with serum-free 1640 and supplemented with serum-free 1640 to a volume of 200 ul. Then 800ul of 1640 medium containing 20% fetal bovine serum was added to the 24-well plate. The chambers were placed into the 24-well plate and incubated for 28–32 h. At the end of incubation, the chambers were removed and fixed in methanol and stained with aqueous crystal violet solution. Finally, randomly selected fields of view were photographed (100X, Olympus, Tokyo, Japan) and counted for statistical purposes. Statistical analysis was performed with GraphPad Prisme 8.

### Clonal efficiency assay

Cells (1000 per well) were seeded in 6-well plates, grown overnight, and then irradiated with 6 Gy of X-ray. After irradiation, cells were grown for two weeks. Cell clones were fixed and stained with crystal violet. The stained cells and colonies (> 50 cells/colony) were photographed and counted. The relative *clone formation* ability was calculated as ability % = (mean experimental *clone* number/mean control *clone* number) × 100. The survival rate of clonogenic cells was used to calculate the sensitization enhancement rate (SER) induced by CD155 knockdown. Survival curves were obtained using GraphPad Prism 8.0. Mean lethal dose (D0) and quasi-threshold dose (Dq) were derived by fitting survival curves to a single-shot multi-target model (y = 1-[1-e(^−kx^)]^N^). SER of D0 (SER_D0_) and Dq (SER_Dq_) were used to assess radioresistance.

### Immunohistochemistry (IHC)

EC tissue was fixed with 10% formalin, embedded in paraffin and then cut into 3 μm sections. Sections were dried at 80 °C for 15 min, dewaxed in xylene, rinsed in ethanol at various concentrations, and rehydrated in double-distilled water. Antigens were unmasked by microwaving the sections in 10 mmol/L of citrate buffer (pH 6.0) for 15 min. The sections were then incubated with hydrogen peroxide to block peroxidase and incubated overnight at 4 °C with anti-CD155 antibody (1:200; Abcam, ab267788). Negative controls were incubated with PBS instead of the primary antibody. The sections were then incubated with horseradish peroxidase (HRP)-labeled streptavidin and the biotinylated secondary antibody. The sections were stained with DAB and counterstained with hematoxylin. We selected sections randomly (×200 magnification) and invited two pathologists to evaluate and score themindependently. The intensity of the dye color and number of positive cells were used to assess the scores. The intensity was graded as 0 (no staining), 1 (weak staining), 2 (moderate staining), and 3 (intense staining). On the other hand, the percentage of positive cells was divided into four categories: 0 (5%), 1 (5–25%), 2 (25–50%), 3 (51–75%), and 4 (> 75%). The final score was the product of these two scores. If the multiplied score was more than 8, the degree of expressiveness was considered as “high”, else it was characterized as “low.“

### In vivo experiments

 All animal procedures were performed according to the protocol approved by the Ethics Committee of Qilu Hospital of Shandong University. For this study, female nude mice were strictly fed according to the guidelines of the institution. For the tumor xenograft model of EC, female NU/NU nude mice were randomly divided into six groups of five mice each, and labeled according to the type of cells and treatment they were subjected sh-NC (xenografted with cells expressing the shControl), sh-CD155 (xenografted with cells expressing the shCD155), sh-NC + R (xenografted with cells expressing the shControl and subjected to radiation after), sh-CD155 + R (xenografted with cells expressing the shCD155 and subjected to radiation after), OE-NC (xenografted with cells overexpressing the control construct), and OE-CD155 (xenografted with cells overexpressing CD155). For xenografting, cells were resuspended in saline and subcutaneously injected into the groin region of nude mice. The experimental nude mice were anesthetized and exposed to X-rays. The sh-NC + R and sh-CD155 + R groups were administered 6 Gy radiation 10, 12, and 14 days after injection. Tumor volume (V) was measured every three days in two dimensions (a, b; a > b) and calculated according to the following formula: V = a×b²/2. The mice were sacrificed 28 days after injection. In metastasis mouse models, twelve 4-week-old female NU/NU nude mice were randomly divided into four groups, each containing three mice. CD155 was knocked down and overexpressed in GFP-tagged Eca109 cells. The cells were injected intravenously into nude mice (n = 3). The mice of the metastasis mouse model were sacrificed 60 days after injection. Eca109 expressing GFP xenograft growth was pictured with bioluminescent imaging using an In Vivo Imaging System (IVIS) Spectrum (Perkin-Elmer; Waltham, MA). The lungs and the livers of the mice also underwent bioluminescence imaging.

### Hematoxylin-eosin (HE) staining

HE staining was performed according to a general protocol. In short, after dewaxing and rehydration, the tissue sections were incubated with hematoxylin solution (ZSGB-BIO, China) for 5 min and fractionated in 1% acid alcohol solution (1% hydrochloric acid in 75% ethanol), and then rinsed with distilled water. The sections were then stained with the eosin solution (ZSGB-BIO, China) for 2 min, followed by dewatering in graded alcohol and clearing in xylene. Finally, the tissue sections were photographed under a microscope.

### Immunofluorescence (IF)

Cells were grown overnight on coverslips and fixed with methanol. After washing 3 times with PBS, the cells were permeabilized with 0.2% Triton X100 in PBS and closed with goat serum for 1 h at room temperature. The coverslips were then incubated with YAP (14074T) antibody overnight at 4 °C, followed by incubation with fluorescently coupled secondary antibody for 1 h at room temperature. After washing 3 times with PBS, the slides were stained with DAPI for 15 min, followed by washing with PBS.Finally, the coverslip is fixed to the slide with anti-fluorescence quencher reagent. The coverslip is fixed to the slide with anti-fluorescence quencher reagent. The coverslip is fixed to the slide with ProLong Gold Antifade reagent. The slides were then examined with microscope and the YAP was analyzed for fluorescence intensity by ImageJ software (n = 3).

### Statistical analysis

For cell experiments, data are presented as the mean ± SD and were analyzed by a two-tailed unpaired t-test using Prism 8.0. The interaction between radiation and CD155 knockdown was analyzed using the Bliss independence model [22]. Based on the inhibition rate (IR), the combined percentage inhibition, IR_R+sh−CD155_, was predicted using the following formula: IR_R+sh−CD155_ = IR_R_+IR_sh−CD155_−IR_R_×IR_sh−CD155_, where IR_R_ and IR_sh−CD155_ were the IR of mono-treatment with radiation and sh-CD155, respectively. Then, the actual combined percentage inhibition, IR_R+sh−CD155_, was compared with IR_R+sh−CD155(Thero.)_. IR_R+sh−CD155_>, =, and < IR_R+sh−CD155(Thero.)_ indicated synergistic, additive, and antagonistic effects, respectively. Overall survival (OS) and progression-free survival (PFS; the time from treatment to disease progression or death from any cause) were assessed using data represented as mean ± standard deviation. GraphPad Prism 8 was used for statistical and survival analyses. Survival analysis *p-*values were based on log-rank tests. Statistical significance was defined when *p* < 0.05.

### Analysis of human PC datasets

Data analysis was performed using the cBioPortal Cancer Genomic and Oncomine websites.

## Results

### CD155 overexpression correlates with poor prognosis in EC

**Fig. 1:**
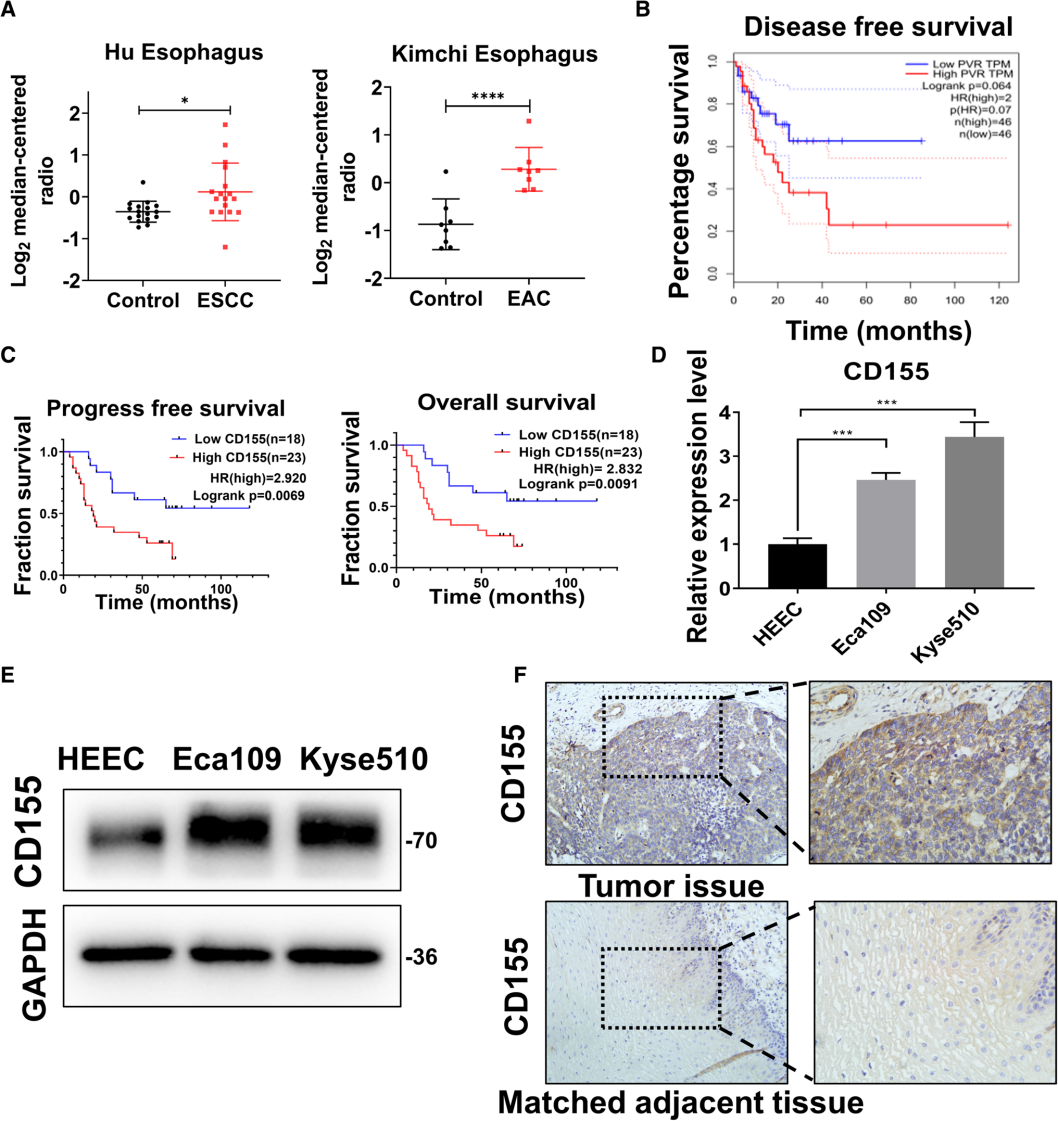
CD155 overexpression is correlated with poor prognosis in EC. **A** Analysis of CD155 protein levels in esophageal squamous cell carcinoma (ESCC) and esophageal adenocarcinoma (EAC) using gene expression data available in Oncomine. Expression levels of CD155 in normal (left plot) and cancer tissue (right plot) were plotted using GraphPad Prism software. The y-axis represents median ratio. **B** Progression-free survival (PFS) analysis in patients with high and low CD155 expression levels. Image obtained from the cBioPortal. **C** Kaplan-Meier analysis of survival in patients with ESCC with low and high CD155 expression. **D**, **E** Analysis of CD155 expression levels in HEEC, Eca109 and Kyse510 cultured cells. **D** mRNA levels were analyzed using qRT-PCR. **E** Protein levels were measured using western blot **F** Immunohistochemistry (IHC) analysis of CD155 protein levels in ESCC tissues and adjacent normal tissues. Data are expressed as mean ± SD. *p < 0.05, ** *p* < 0.01 and *** *p* < 0.001

To assess the differential expression of CD155 in the para-tumorous and tumor tissues, we analyzed CD155 protein levels in different EC and control tissues using publicly available data in Oncomine. We could observe that CD155 is expressed in higher levels in EC tissues than in normal gastric mucosa (Fig. 1A). Additionally, data from cBioPortal showed reduced PFS in patients with high CD155 expression (Fig. 1B). Moreover, we analyzed the OS and PFS rates of postoperative patients with EC using the Kaplan-Meier method, plotted survival curves for the different CD155 expression groups, and used the log-rank test to analyze differences between those groups. We found that the OS and PFS rates were lower in the group with high CD155 expression than in those with low or no CD155 expression (Fig. 1C). These data suggest the existence of a co-relation between EC, higher CD155 protein levels and lower OS and PFS rates in patients. To further validate the effects of differential expression of CD155, we confirmed that EC cells (Eca109, Kyse510) express higher CD155 levels of both mRNA and protein than human esophageal epithelial cells (HEECs) (Fig. 1D, E). In addition, IHC analysis of EC tissues and normal adjacent tissues revealed that CD155 levels were higher in cancerous esophageal tissues than in normal neighboring tissues (Fig. 1F). These results suggest that CD155 expression is significantly higher in patients with esophageal cancer than in normal controls, and that high expression of CD155 is associated with a poor prognosis in patients.

### CD155 expression levels in EC are upregulated after X-ray irradiation

**Fig. 2:**
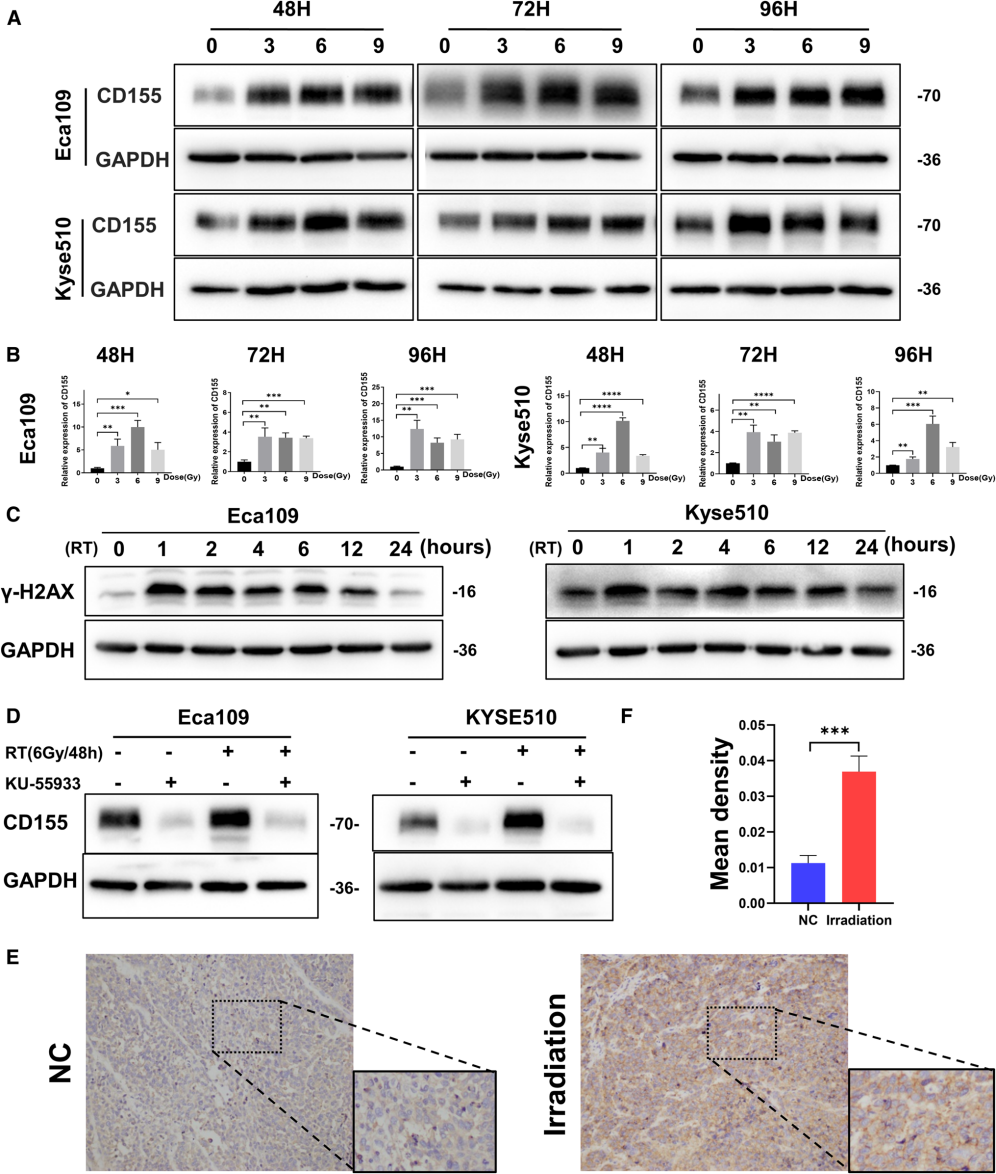
CD155 expression levels in EC are upregulated after X-ray irradiation. **A**, **B** Analysis of CD155 expression levels in Eca109 and Kyse510 cultured cells after X-ray irradiation. **A** mRNA levels were analyzed using RT-PCR. **B** Protein levels were measured using western blot analysis. **C** Changes in the expression of γ-H2AX were detected by western blotting when the esophageal cancer cell lines were irradiated at 6 Gy within 24 h. **D** The expression of CD155 was detected via western blotting after the addition of the ATM inhibitor, KU55933. **E** Immunohistochemistry (IHC) analysis of CD155 protein expression in tumors of irradiated and non-irradiated nude mice. **F** Statistical analysis of IHC results presented in **E**. Data are expressed as the mean ± SD. *p < 0.05, ** p < 0.01 and *** p < 0.00 1

To assess the effect of irradiation over EC, Eca109 and Kyse510 cells were exposed to different X-ray doses combined with different exposure times. RT-PCR and western blot analyses were then used to detect the mRNA and protein levels of CD155 in each experimental condition. We found that the expression of CD155 in EC cells was significantly higher after x-ray irradiation (Fig. 2A, B).

We then examined the irradiated EC cell line for the DNA damage marker γ-H2AX via western blotting. We found that γ-H2AX expression in the irradiated EC cell line gradually decreased after reaching a certain peak (Fig. 2C). This indicated that the cells underwent radiation damage to DNA. In addition, we treated the cells with KU-55933, an inhibitor of ATM, a marker related to DNA damage repair, and irradiated them. Western blotting results showed that the elevated expression of CD155 induced by irradiation was significantly suppressed (Fig. 2D). This suggested that the elevation of CD155 after irradiation in EC was caused by DNA damage repair after DNA damage caused by X-ray irradiation.Additionally, irradiation of nude mice with xenografted tumor showed that t*he expression* levels of CD155 of the tumor markedly increased after irradiation (Fig. 2E, F). Taken together, these data indicate that tumor X-ray radiation induces relatively increase in CD155 expression levels, both in vitro and in vivo.

### Expression of CD155 affects the proliferation and migration of EC

**Fig. 3:**
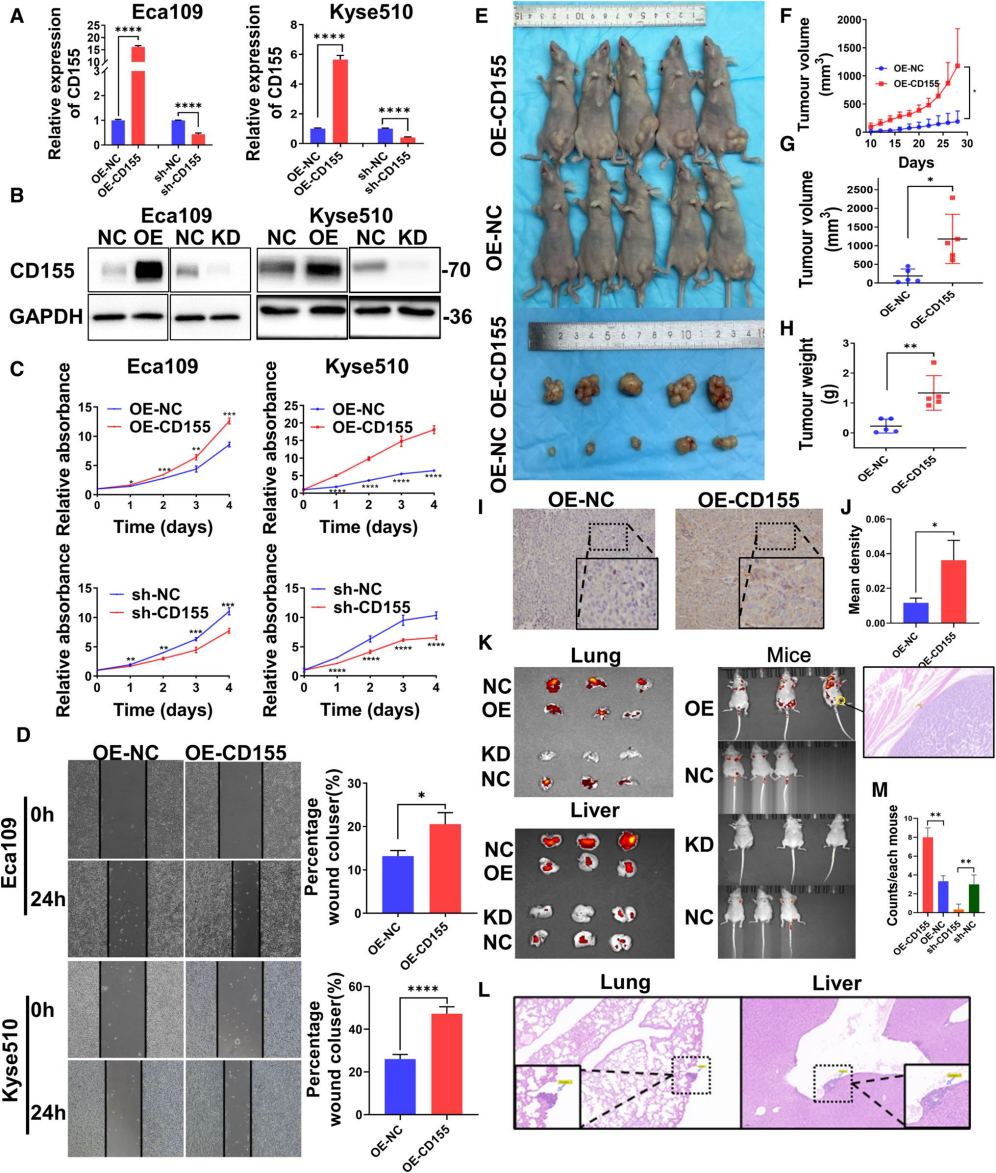
Expression of CD155 affects the proliferation and migration of EC. **A**, **B** CD155 expression in stably transfected cells. **A** RT–PCR was used to analyze mRNA levels. **B** Western blot revealed CD155 protein levels. **C** Cell viability was analyzed using the CCK8 assay. **D** Migration rates were measured using the wound-scratch assay method. Left: Image of one wound-scratch assay. Right: Quantitative analysis of the wound-scratch assays performed. **E** Image of xenografted nude mouse model and respective xenografted tumor. **F** Tumor volume curves of xenograft tumors overexpressing CD155 and NC (n = 5 for each condition). **G**, **H** analysis of xenograft tumors on the day of tumor removal. The volume (**G**) and weight (**H**) of each tumor was measured. **I**, **J** Immunohistochemistry (IHC) analysis of CD155 protein expression in the tumor tissues of nude mice. **I** Images of the tissues. **J** quantitative analysis of the images was performed using Image Pro Plus, and statistical analysis was performed using GraphPad Prism 8.0. **K**, **L** CD155 was knocked down and overexpressed in GFP-tagged Eca109 cells. The cells were injected intravenously into nude mice (n = 3). Two months later, the mice and the lungs and liver of the mice underwent bioluminescence imaging. **K** HE staining of leg metastasis (100 X magnification). **L** Representative images of HE staining of the lung and liver metastases (100 X magnification). Scale bar, 100 μm. (M) Showing the statistical analysis for the counts of metastases imaged in vivo in nude mice in Fig. 3K. *, ** *p* < 0.05, *** *p* < 0.001, **** *p* < 0.0001

**Fig. 4:**
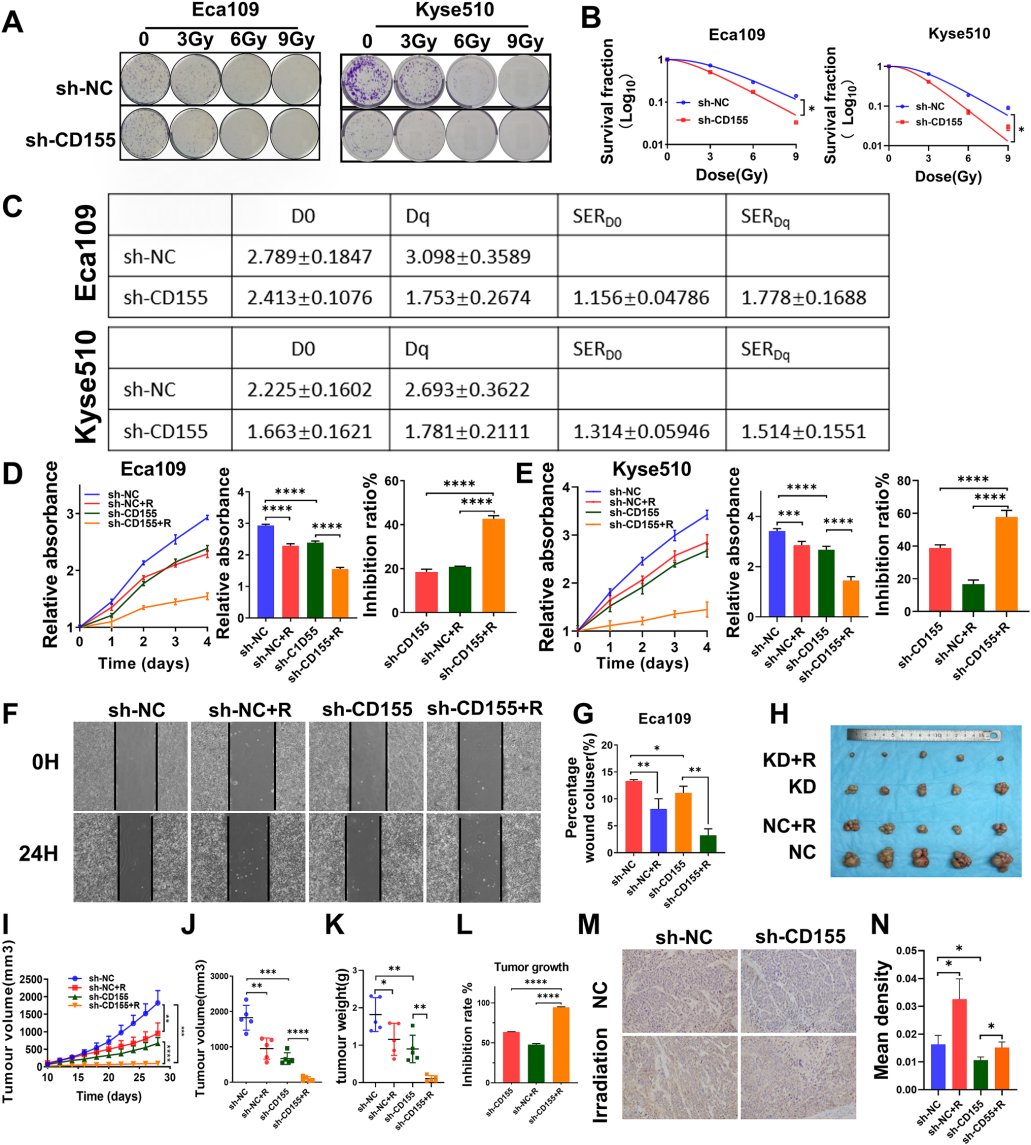
Knockdown of CD155 enhances radiosensitivity of EC. **A** Colony formation analysis of Eca109 and Kyse510 cells stably expressing sh-CD155 or sh-NC after exposure to 0, 3, 6, and 9 Gy of radiation. **B** Survival curves were obtained based on the clonal efficiency assay. Data are presented as the mean ± SD of independent experiments (n = 3). **C** D0, Dq, SER_D0_, and SER_Dq_ were calculated using the single-hit multitarget model. **D**, **E** Eca109, and Kyse510 cells expressing sh-NC or sh-CD155 were exposed to radiation, and cell viability was analyzed using the CCK8 assay. Left: CCK8 assay results; middle: statistical analysis of CCK8 assay; right: IR of cell viability was calculated and presented as mean ± SD (n = 3). **** P < 0.0001, two-tailed unpaired t-test. **F** wound-scratch assays were imaged immediately after the initial scratch (T0) and 24 h later. **G** Quantification of the relative cell migration in the wound-scratch assay. **H**–**L** Tumors from different groups (sh-NC, sh-NC + R, sh-CD155, and sh-CD155 + R) were xenografted in nude mouse and collected 28 days later. **H** Tumor images. **I** Tumor growth curve in nude mice, p-values were calculated using a two-tailed unpaired test. **J** Tumor volumes were recorded every three days and are expressed as the mean ± standard deviation (n = 5). **K** Tumor weight was measured in each case. **L** The IR on xenograft growth was calculated based on the tumor volume and shown as mean ± SD (n = 5). *, ** *p* < 0.05, *** *p* < 0.001, **** *p* < 0.0001, two-tailed unpaired t-test. **M** Representative IHC images showing CD155 in xenografts from Eca109 cells (magnification, ×100). **N** Statistical analysis for metastasis in nude mice shown in (**M**). *, ** p < 0.05, *** p < 0.001, **** p < 0.0001

To investigate more in detail the effect of CD155 expression on EC cells, different Eca109 and Kyse510 derived cell lines were created: cells stably overexpressing CD155 (Eca109_OE-CD155 and Kyse510_OE-CD155) and cells stably expressing a short hairpin RNA for CD155 (sh-CD155) that promotes specific CD155 mRNA degradation and consequently, gene expression knockdown (Eca109_sh-CD155 and Kyse510_sh-CD155) (Fig. 3A, B). Control cell lines for overexpression (Eca109_OE-NC and Kyse510_OE-NC) and for gene knockdown (Eca109_sh-NC and Kyse510_sh-NC) were also created. Taking advantage of these tools, we conducted different in vivo and in vitro tests to address the effect of CD155 levels in EC cells. CCK8 assay analysis revealed that CD155 overexpression promoted cell proliferation (Fig. 3C), as measured by the higher cell count of Eca109_OE-CD155 and Kyse510_OE-CD155 populations, when compared with Eca109_OE-NC and Kyse510_OE-NC cells, respectively. Moreover, the lower cell number observed in Eca109_sh-CD155 and Kyse510_sh-CD155 cell populations suggests that inhibition of CD155 significantly inhibited cell growth (Fig. 3C). Additionally, the wound-scratch assay demonstrated that overexpression of CD155 promoted the migration of EC cell lines (Fig. 3D), when compared with control cells, while the knockdown of CD155 reduced EC cell migration (Fig. 4F). In addition, we also performed transwell assay to verify the effect of CD155 expression on the migration of EC cells. Results revealed that EC cells with high CD155 expression had increased migration, while the migration of the CD155 knockdown group was reduced (Additional file 1: Figure S1 A, B).

To validate the in vitro results, we tested how knockdown and overexpression of CD155 affected the formation of subcutaneous xenograft tumors in nude mice. Nude mice were injected with either Eca109_OE-CD155 or Eca109_sh-CD155 cells. We could observe that the mean volume and weight of xenograft tumors induced with Eca109_OE-CD155 cells was significantly higher than those induced with Eca109_OE-NC cells (*p* < 0.05) (Fig. 3E-H). Likewise, unlike the Eca109_sh-NC cell line, Eca109_sh-CD155 cells have a reduced ability to form tumors (*p* < 0.05) (Fig. 4 H-L).

To further explore the ability of CD155 to influence the migration *of* EC cells in vivo, Eca109_OE-CD155, Eca109_sh-CD155 cells or the respective control cells (Eca109_OE-NC and Eca109_sh-NC) were injected into the tail vein of nude mice. Strikingly, mice receiving Eca109_OE-CD155 cells showed lung and liver metastases, whereas mice transplanted with Eca109_sh-CD155 cells had much lower metastasis levels (Fig. 3K). Figure 3M shows the statistical analysis of metastasis in nude mice. The results revealed that metastasis was higher in CD155-overexpressing nude mice than in that in the control group, while the opposite result was obtained for the CD155-knockdown nude mice. HE staining of the metastasis mouse models revealed lung and liver metastases in nude mice (Fig. 3L).

Taken together, our data suggest that CD155 expression promotes EC cell proliferation and migration. In contrast, CD155 knockdown inhibits EC proliferation and migration.

### Knockdown of CD155 enhances radiosensitivity of EC

Since irradiated EC cells had higher expression of CD155, we investigated whether CD155 levels can impact EC radiosensitivity. We analyzed the radiosensitivity of EC cell lines using the clonal efficiency assay (Fig. 4A) and constructed survival curves for each of the conditions. Clonogenesis assays and survival curves showed that Eca109_sh-CD155 cells had a lower clonal efficiency ability after radiation, when compared to Eca109_sh-NC cells (Fig. 4B). The analysis of the survival curves showed that Eca109_sh-CD155 cells had a lower D0 (2.413 ± 0.1076) than Eca109_sh-NC cells (2.789 ± 0.1847) (Fig. 4C). Additionally, Eca109_sh-CD155 cells showed a lower Dq than Eca109_sh-NC cells (2.413 ± 0.1076). We could observe that CD155 knockdown resulted in SER_D0_ and SER_Dq_ levels of 1.156 ± 0.04786 and 1.778 ± 0.1688, respectively. The Kyse510 cell line showed similar results (Fig. 4A-C): D0 (2.225 ± 0.1602) and Dq (2.693 ± 0.3622) were higher in the Kyse510_sh-NC cells than in the Kyse510_sh-CD155 cells. SER_D0_ and SER_Dq_ levels were 1.314 ± 0.05946 and 1.514 ± 0.1551 in Kyse510_sh-CD155 cells. Our data *show* that knockdown of CD155 increased the sensitivity of EC cells to radiotherapy. We also analyzed the combined impact of CD155 knockdown and radiation, using the CCK8 assay (Fig. 4D, E). The Bliss independence model showed that radiation and CD155 knockdown have synergistic effects in the inhibition of cell viability (Table 2). Also, in wound-scratch experiments, we observed that the combination of CD155 knockdown and irradiation showed the strongest effect in the reduction of EC cell migration, when compared with the other conditions (Fig. 4F, G). We observed the same results in the transwell assay, where X-ray irradiation inhibited the migration of cells in the knockdown CD155 group more significantly (Additional file 1: Figure S1C, D).

**Table 2:** Effects of irradiation and CD155 knockdown combination on cell viability and xenografts growth

	Cell viability/tumor growth inhibition rate (IR)%				Difference	Effects
	IR_R_	IR_sh−CD155_	IR_R+sh−CD155_	IR_R+sh−CD155(Theor.)_	IR_R+sh−CD155_ -IR_R+sh−CD155(Theor.)_	
Eca109	20.83	18.48	42.66	35.46	7.20	Synergy
Kyse510	16.68	38.79	57.75	48.96	8.79	Synergy
Murine Xenografts	47.83	63.59	94.50	81.00	13.50	Synergy

To further investigate the effect of CD155 on the radiosensitivity of EC, nude mice were subcutaneously implanted with either Eca109_sh-CD155 or Eca109_OE-CD155 cells. When tumors reached a volume of 200 mm^3^, the mice were irradiated with 6 Gy of X-rays every three days. Tumors were removed 28 days after the first irradiation. As shown in Fig. 4H-K, sh-CD155 + R mice had lower tumor volume and weight than sh-NC + R mice. The Bliss model also revealed that irradiation and CD155 knockdown synergistically reduced tumor development (Fig. 4L, Table 2). Xenograft immunostaining for CD155 showed that the expression of CD155 was significantly decreased in sh-CD155 transfected tumors, and CD155 was markedly increased in the irradiated group (Fig. 4M).

### CD155 stimulates EC cellular proliferation and migration through the Hippo signaling pathway

**Fig. 5:**
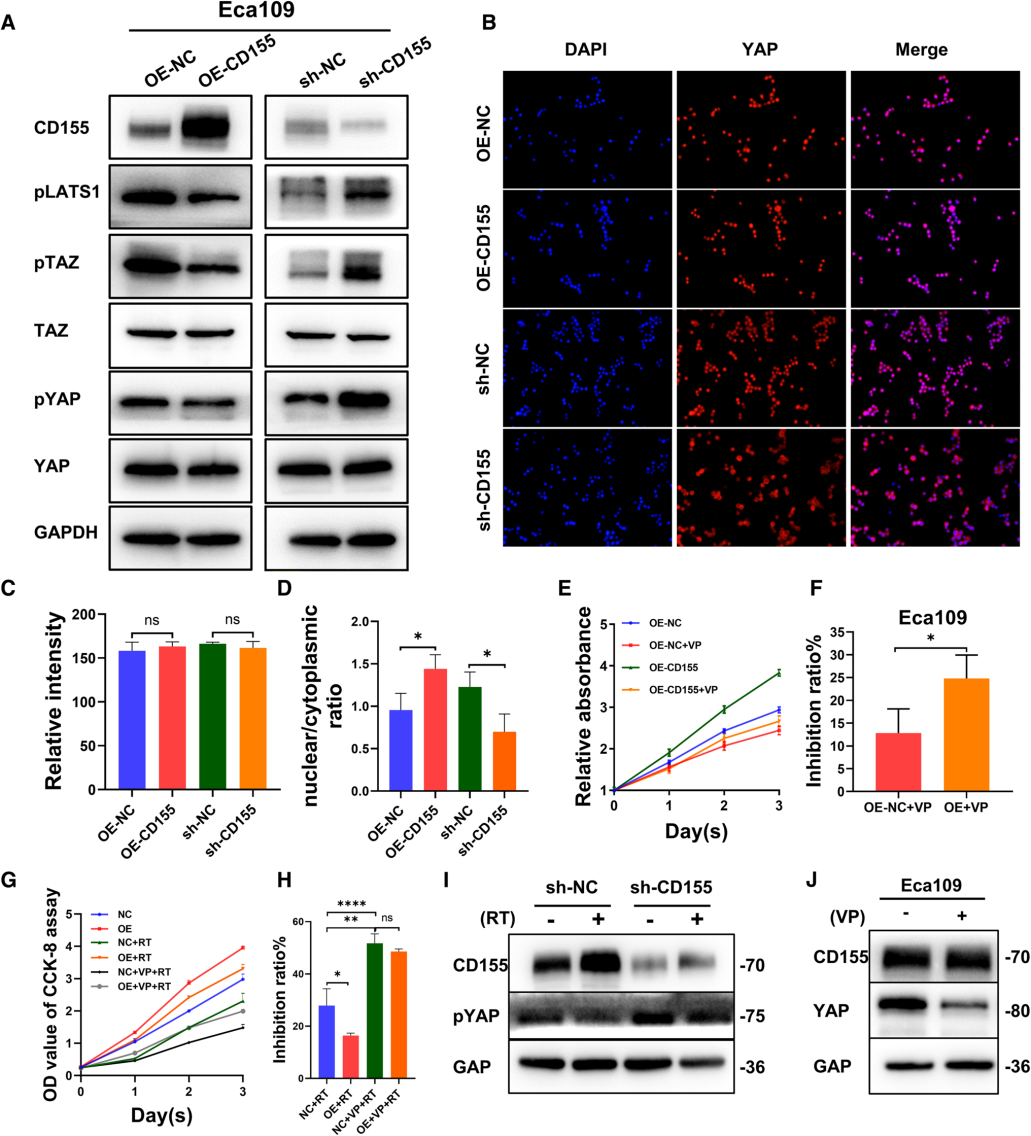
CD155 stimulates EC cellular proliferation and migration through the Hippo signaling pathway. **A **Western blot analysis of CD155, pLATS1, pTAZ, TAZ, pYAP, and YAP in cells overexpressing or depleted of CD155. **B** Immunofluorescence analysis of YAP in cells overexpressing or depleted of CD155. red, YAP; blue, DAPI. **C** Quantification of immunofluorescence staining using ImageJ software. **D** Analysis of the nucleoplasmic ratio of **B** using ImageJ. **E**, **F** CCK8 analysis showed that verteporfin treatment reversed the pro-proliferative effect of CD155 overexpression in EC cells. **G**, **H** CCK-8 assay was performed to examine the effect of verteporfin (VP) in combination with irradiation on the proliferative capacity of esophageal cancer cell lines. **I** WB was performed to detect the expression levels of p-YAP and CD155 in esophageal cancer cells after irradiation. **J** WB for the effect of VP on CD155 and YAP

CD155 regulates the radiosensitivity of EC, so it is particularly important to find its regulatory pathways. Some studies have shown that the transcriptional factor YAP of the Hippo signaling pathway regulates EC cell proliferation and migration. Moreover, Zhang et al. has been suggested that YAP may be involved in the radioresistance of EC. Taking this into consideration, we analyzed the expression of various Hippo pathway proteins in the Eca109 cell lines. Western blot analysis showed that overexpression of CD155 induces a decrease in the levels of inactivated (phosphorylated) TAZ and YAP in Eca109 cells, whereas knockdown of CD155 (Eca109_sh-CD155 cells) resulted in the opposite effect, i.e., in higher levels of inactivated TAZ and YAP. In addition, we could observe that the total protein levels of YAP and TAZ remained unchanged (Fig. 5A). These data suggest the existence of a positive correlation between CD155 overexpression and the inactivation of the Hippo pathway. Immunofluorescence analysis was used to identify the nuclear (active) or cytoplasmic (inactive) localization of YAP. YAP was found mainly in the nucleus of Eca109_OE-CD155 cells, while in Eca109_sh-CD155 cells, YAP was primarily found in the cytoplasm (Fig. 5B). ImageJ analysis also showed no change in the total amount of YAP in the cell (Fig. 5C). Statistical results showed that YAP in CD155-overexpressing Eca109 cells was mostly localized in the nucleus, while the opposite was true for the CD155 knockdown group (Fig. 5D). As a result of the CCK-8 assay (Fig. 5E, F), we found that the proliferative capacity of Eca109 EC cells overexpressing CD155 was significantly more suppressed after inhibition with verteporfin (VP), an inhibitor of YAP, than in the control group. We then performed a combination of irradiation and verteporfin treatment on EC cells. We also performed a CCK-8 assay divided using NC, NC + radiotherapy (RT), NC + VP + RT; OE, OE + RT, and OE + VP + RT groups (Fig. 5F, G) and found that EC cells overexpressing CD155 showed greater resistance to irradiation compared to the control group. The sensitivity of EC cells overexpressing CD155 to irradiation was improved after inhibition with VP, with no statistically significant difference compared with the control group. This suggested that CD155 regulated the radiosensitivity and proliferation ability of EC through YAP. In addition, after irradiating EC cells, we examined the expression levels of phosphorylated YAP and CD155, key molecules in the Hippo pathway, via western blotting (Fig. 5H). It was found that the expression levels of CD155 and phosphorylated YAP both changed accordingly in irradiated cells, with a decrease in the expression of phosphorylated YAP when the expression of CD155 was elevated. The effect of VP on the expression levels of CD155 and YAP in EC we examined via western blotting (Fig. 5J). The results also suggested that there was a radiation-induced elevation of CD155 expression that inhibited Hippo pathway activation.

## Discussion

CD155 is an adhesion molecule that belongs to the Nectin-like family. It is involved with a variety of cancer-associated functions, including the intrinsic activity of tumor cells that regulate proliferation, adhesion, and migration, as well as the ability to influence the immune response by binding to the immunomodulatory receptors dNaM-1, CD96, and TIGIT [23]. During recent years, CD155 expression has been extensively reported in tumor tissues and stroma. Recent studies have shown that CD155 is expressed at low levels in various normal cells, including endothelial cells, epithelial cells, nerve cells, and fibroblasts [24]. It was additionally shown that CD155 is extensively expressed in a variety of malignancies [11, 25–29], and its protein levels are strongly correlated with tumor development and poor prognosis [24, 28, 30, 31]. These results suggest that CD155 can be a good biomarker for cancer progression and prognosis assessment. However, the mechanism of CD155 activity in EC was not yet investigated. In this study, analysis of clinical specimens and the Oncomine dataset showed that CD155 protein expression was higher in EC patients, when compared with healthy controls. Moreover, we found that CD155 expression was higher in EC tissues than in adjacent tissues. We also observed that EC cell lines (Eca109 and Kyse510) expressed more CD155 than HEEC cells. Our assays showed that overexpression of CD155 promoted EC progression both in vivo and in vitro. Notably, these observations are consistent with those reported for other tumor tissue types [32]. Moreover, IHC analysis showed that high CD155 expression negatively correlated with OS and PFS in EC patients, suggesting that CD155 expression plays a role in the genesis and treatment response of ECs.

Baseline clinical staging remains a significant concern for oncologists, when planning the treatment of patients with EC, and tumor heterogeneity is usually not considered. Even when individuals are diagnosed at the same stage, the treatment results differ significantly. Therefore, personalized approaches to the diagnosis and treatment of patients with EC are urgently needed. Although radiotherapy is known to be an effective non-surgical treatment for EC [33, 34], its efficacy is severely limited by the radioresistance of tumor cells. Acquired radioresistance during radiation is a significant problem for patients with EC. Although several pathways to reverse tumor radioresistance in EC have been studied (Ex.: CAF-promoted DNM3OS) [35], the molecules to predict radioresistance are largely unknown, and novel methods to sensitize cells to irradiation are urgently needed. This study focused on the identification of a biomarker to predict radiotherapy efficacy and identify appropriate patients most likely to benefit from radiation. In our investigation, we discovered for the first time that radiation induces and increase in CD155 protein levels in ECs, both in vivo and in vitro, being also associated with poor prognosis. This suggests that CD155 is a potential target for improving radiosensitivity during clinical treatment. Furthermore, we found that CD155 knockdown increased the sensitivity of EC to radiation therapy both in vitro and in vivo. Our data imply that CD155 plays an important role in radiation resistance, and targeting CD155 may be a suitable therapeutic strategy for treating EC.

Yorkie (ortholog of YAP/TAZ) was discovered to be an effector of the Hippo signaling system in Drosophila, and the involvement of YAP/TAZ in tissue homeostasis maintenance has consequently been extensively explored in human cells [36]. In particular, activated YAP/TAZ leads to increased tumorigenicity, proliferation, drug resistance, epithelial-to-mesenchymal transition (EMT), and metastasis in a variety of tumor cell types, all of which have an associated poor prognosis in patients [37]. Although some studies analyze the association between the Hippo pathway and resistance to radiotherapy in EC, its relationship with CD155 was never investigated. The Hippo signaling pathway is a kinase cascade that regulates organ size by co-regulating cell proliferation and death [36, 38–41]. Previous studies have found that activation of the Hippo signaling pathway promotes ESCC cell proliferation, migration, and invasion and inhibits apoptosis [42, 43]. YAP exhibits anti-radiation effects on gliomas by promoting DNA damage repair [41, 44]. Previous studies found that because CD155 upregulation is related to DNA radiation damage, DNA damage or DNA replication inhibitors induce CD155 expression through activation of ATM, as well as ATM- and rad3-related (ATR) protein kinases [45, 46]. Radiation therapy causes DNA damage through direct or indirect effects [47–49]. In addition, Zhang et al. indicated that radioresistance in ESCC may be associated with the Hippo pathway [50]. Taking all this into account, we speculated that CD155 could regulate radiosensitivity of esophageal cancer through Hippo pathway. We used verteporfin, an inhibitor of YAP, which produced a stronger inhibitory effect on the proliferation EC cell lines with high CD155 expression thanthe negative control. Verteporfin inhibited the proliferation of the EC cells caused by the elevated expression of CD155. It also improved the radioresistance caused by elevated CD155 expression. Radiation-induced elevated CD155 expression in EC mediates cancer progression and radioresistance through the Hippo-YAP pathway. This suggests that CD155 blockade may be a new research direction to improve the efficacy of radiation therapy for EC with high CD155 expression. Targeting CD155 and YAP may be an effective way to improve the radiosensitivity of EC.

## Conclusions

In conclusion, CD155 expression is increased in some patients with EC, which is associated with a poor prognosis of EC. Furthermore, irradiation of EC cells induced an increase in CD155 protein levels and CD155 promoted proliferation and migration. Moreover, CD155 knockdown increased the radiosensitivity of ECs by regulating the Hippo-YAP pathway. These data suggest that CD155 is a potential sensitizing target for radiotherapy of EC. Furthermore, CD155 acts as a ligand for the immunomodulatory receptor TIGIT, suggesting that our studies provide strong evidence for combined immune and radiation therapy.

## Supplementary Information

**Additional file 1: Figure S1. A-D **Transwell assay for cell migration ability.

## Data Availability

All the datasets generated and analyzed in the present study are available from the corresponding author on reasonable request.

## Abbreviations

EC: Esophageal cancer
ESCC: Esophageal squamous cell carcinoma
EAC: Esophageal adenocarcinoma
IHC: Immunohistochemistry
VP: Verteporfin
IR: Inhibition rate
