# Supplementary figures and images for: CD155 promotes radioresistance and malignancy of esophageal cancer by regulating Hippo-YAP pathway
Source: Discov Oncol. 2022 Jun 29;13:53. doi: 10.1007/s12672-022-00515-z (PMC9243211; doi:10.1007/s12672-022-00515-z)

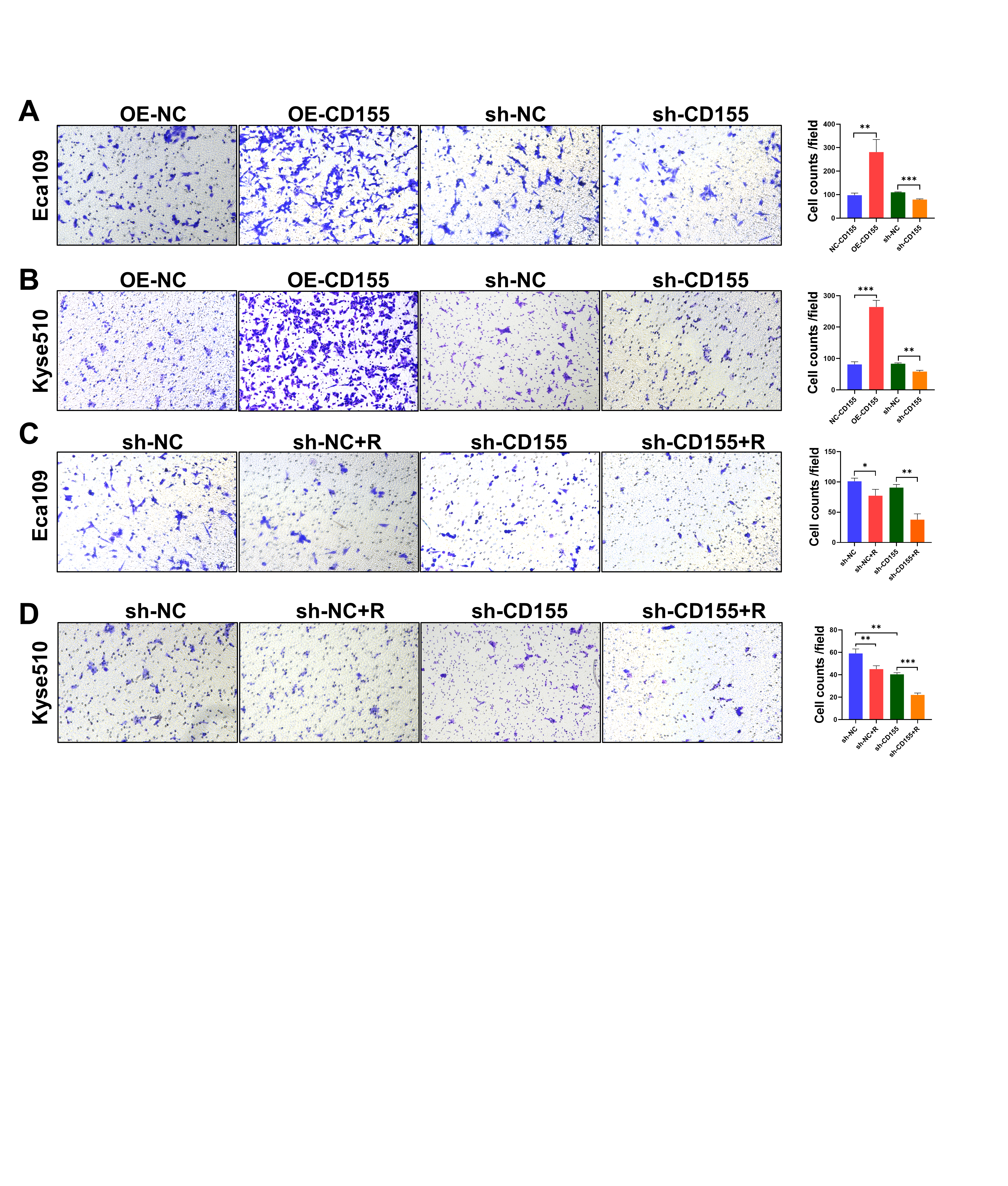

Supplement: Supplementary file 1 — Additional file 1: Figure S1. A-D Transwell assay for cell migration ability. [file 12672_2022_515_MOESM1_ESM.tif]
